# The impact of 4D-Flow MRI spatial resolution on patient-specific CFD simulations of the thoracic aorta

**DOI:** 10.1038/s41598-022-19347-6

**Published:** 2022-09-06

**Authors:** Molly Cherry, Zinedine Khatir, Amirul Khan, Malenka Bissell

**Affiliations:** 1 CDT in Fluid Dynamics, School of Computing, University of Leeds, Leeds, LS2 9JT UK; 2 School of Engineering and the Built Environment, Birmingham City University, Birmingham, B4 7XG UK; 3 School of Mechanical Engineering, University of Leeds, Leeds, LS2 9JT UK; 4 School of Civil Engineering, University of Leeds, Leeds, LS2 9JT UK; 5 School of Medicine, University of Leeds, Leeds, LS2 9JT UK

**Keywords:** Biomedical engineering, Computational models

## Abstract

Magnetic Resonance Imaging (MRI) is considered the gold standard of medical imaging technologies as it allows for accurate imaging of blood vessels. 4-Dimensional Flow Magnetic Resonance Imaging (4D-Flow MRI) is built on conventional MRI, and provides flow data in the three vector directions and a time resolved magnitude data set. As such it can be used to retrospectively calculate haemodynamic parameters of interest, such as Wall Shear Stress (WSS). However, multiple studies have indicated that a significant limitation of the imaging technique is the spatiotemporal resolution that is currently available. Recent advances have proposed and successfully integrated 4D-Flow MRI imaging techniques with Computational Fluid Dynamics (CFD) to produce patient-specific simulations that have the potential to aid in treatments,surgical decision making, and risk stratification. However, the consequences of using insufficient 4D-Flow MRI spatial resolutions on any patient-specific CFD simulations is currently unclear, despite being a recognised limitation. The research presented in this study aims to quantify the inaccuracies in patient-specific 4D-Flow MRI based CFD simulations that can be attributed to insufficient spatial resolutions when acquiring 4D-Flow MRI data. For this research, a patient has undergone four 4D-Flow MRI scans acquired at various isotropic spatial resolutions and patient-specific CFD simulations have subsequently been run using geometry and velocity data produced from each scan. It was found that compared to CFD simulations based on a $1.5\,{\text {mm}} \times 1.5\,{\text {mm}} \times 1.5\,{\text {mm}}$, using a spatial resolution of $4\,{\text {mm}} \times 4\,{\text {mm}} \times 4\,{\text {mm}}$ substantially underestimated the maximum velocity magnitude at peak systole by $110.55\%$. The impacts of 4D-Flow MRI spatial resolution on WSS calculated from CFD simulations have been investigated and it has been shown that WSS is underestimated in CFD simulations that are based on a coarse 4D-Flow MRI spatial resolution. The authors have concluded that a minimum 4D-Flow MRI spatial resolution of $1.5\,{\text {mm}} \times 1.5\,{\text {mm}} \times 1.5\,{\text {mm}}$ must be used when acquiring 4D-Flow MRI data to perform patient-specific CFD simulations. A coarser spatial resolution will produce substantial differences within the flow field and geometry.

## Introduction

Magnetic Resonance Imaging (MRI) is frequently regarded as the gold standard of medical imaging^1–3^. The non-ionising and non-invasive imaging technique allows for accurate delineation of the blood vessels of interest, primarily within the chest cavity. 4-Dimensional Flow Magnetic Resonance Imaging (4D-Flow MRI) as an imaging technique has evolved from standard MRI. It incorporates flow encoding in all three spatial directions, resolved with respect to the three dimensions as well as time^3^, meaning flow velocities can be determined allowing the haemodynamics to be thoroughly investigated. To provide data of a representative flow field of a single heartbeat, 4D-Flow MRI acquires imaging data over hundreds of heartbeats before averaging to determine a representative heartbeat. During the acquisition, approximately 70–80% of the patients heartbeats are suitable for use in reconstructing the flow field of a representative heartbeat. It is commonly accepted that 4D-Flow MRI provides a satisfactory representation of the flow field during an average heartbeat of the patient.

Despite having been available for over a decade, 4D-Flow MRI is only now becoming a useful clinical tool due to recent reductions in scan times required^1^. The rich data-sets obtained from 4D-Flow MRI are one of the main benefits of the technique, as numerous physiological parameters can be determined retrospectively that can aid disease monitoring, risk stratification, and individualised treatment planning.

4D-Flow MRI improves the understanding of the blood flow in major blood vessels and can be used for monitoring disease progression. Multiple studies have demonstrated the use of 4D-Flow MRI to assess the impact a diagnosis of Bicuspid Aortic Valve (BAV) had on the Wall Shear Stress (WSS) in the thoracic aorta^4–6^. The use of 4D-Flow MRI allowed the flow field to be assessed accurately and allowed for a greater understanding of the impacts of BAV. It has also been expressed^6^ that the role of 4D-Flow MRI should be expanded, suggesting it may be successful in anticipating vascular pathology, allowing for interventional treatment before clinical manifestation. These findings were echoed by studies where it was demonstrated that 4D-Flow MRI enabled the tracking of the progression of BAV in paediatric patients, and the assessment of the flow field in patients with aortopathy respectively^7,8^. It was concluded that 4D-Flow MRI provided a robust, accurate and reliable method for delineating the flow field, and produced easily repeatable results.

Limitations that are known to impact the accuracy of 4D-Flow MRI are the spatial and temporal resolutions that are available. If temporal resolutions used are too coarse, the parameters will be inadequate to capture the turbulence within the flow, and will likely lead to the peak velocities experienced being underestimated. In addition to potentially inaccurately recording the flow patterns and haemodynamics of the blood vessel, a coarse spatial resolution will also result in inferior image quality, creating uncertainty as to the location of the vessel walls, creating uncertainty in parameters such as WSS. This limitation has been noted by multiple studies^4,6^. The limitations of the spatiotemporal resolution of 2D Phase Contrast Magnetic Resonance Imaging (PC MRI) in the carotid arteries has been investigated^9^. A carotid artery flow phantom was set up and PC-MRI scans taken at thirty spatiotemporal resolution settings (varying spatially between 0.2 mm and 1 mm, and temporally between 9.1 ms and 142.9 ms). It was found that the mean flow and WSS were independent of temporal resolution, whilst peak flow and Oscillatory Shear Index (OSI) were found to be dependent on both spatial and temporal resolution. The impact the spatiotemporal resolution of PC-MRI has on the quantification of blood flow and vessel wall parameters has also been explored^10^; using a high resolution scan ($\sim 1.4\,{\text {mm}} \times 1.4 \,{\text {mm}} \times 1.4\,{\text {mm}}$, 24.2 ms) and a low resolution scan ($\sim 2.8\,{\text {mm}} \times 1.6\,{\text {mm}} \times 3\,{\text {mm}}$, 48.6 ms) it was found that a limited resolution introduced a systematic underestimation of WSS. The impact the spatial resolution and velocity encoding of PC MRI has on the WSS was also investigated, where it was found that WSS was particularly sensitive to spatial resolution, and cannot be assumed to be linearly or monotonically related to the actual WSS values^11^.

As the haemodynamics are known to be sensitive to the spatial resolution of 4D-Flow MRI, it has been recommended that when conducting 4D-Flow MRI scans, a spatial resolution of $\le 2.5\,{\text {mm}} \times 2.5\,{\text {mm}} \times 2.5\,{\text {mm}}$should be used when investigating the aorta and pulmonary artery, whilst a spatial resolution of $\le 3\,{\text {mm}} \times 3\,{\text {mm}} \times 3\,{\text {mm}}$should be used if the whole heart and greater vessels are of interest^3^. It was also recommended that the temporal resolution be as fine as possible. To avoid results that are direction dependent it was also recommended that isotropic voxels be used^3^. The spatial and temporal resolutions of 4D-Flow MRI scans used in a selection of studies (2010–2019) can be seen in Table 1. It can be seen in Table 1 that it is not common practice to implement an isotropic spatial resolution (risking results being direction dependent) with the spatial resolution in the *z* direction being coarser than recommended in some cases and much smaller in other directions^3^.

**Table 1:** Spatial and temporal resolutions of 4D-Flow MRI scans used in recent studies (2010–2019) that have utilised 4D-Flow MRI to acquire haemodynamic data.

Study	Vessel of interest	Temporal resolution (ms)	Spatial resolution (mm$$^{3})$$
Barker et al.^4^	Ascending aorta	38.4–52.5	$1.8{-}2.1 \times 1.8{-}2.1 \times 2.0{-}2.8$
Barker et al.^4^	Ascending aorta	40.8	$2.1 \times 2.1 \times 2.4$
Barker et al.^5^	Ascending aorta	10–30	$0.82{-}1.56 \times 0.82{-}1.56 \times 5.0$
Hope et al.^6^	Ascending aorta	74–77	$1.17\times 1.56 \times 2.6$
Rose et al.^7^	Ascending aorta	37.6–44	$1.23{-}3.46 \times 1.13{-}2.5 \times 1.2{-}3.0$
De Beaufort et al.^8^	Ascending aorta	38–47	$2.0{-}3.0 \times 2.3{-}3.8 \times 3.4{-}5.0$
Biglino et al.^12^	Ascending aorta	33.4	$2.2 \times 2.2 \times 2.2$
Hellmeier et al.^13^	Ascending aorta	$\frac{1}{25}$th of a heartbeat	$1.83{-}2.25 \times 1.83{-}2.25 \times 2.0{-}2.8$
Kimura et al.^14^	Ascending aorta	33	n/a
Kimura et al.^14^	Ascending aorta	43	n/a
Miyazaki et al.^15^	Aortic arch	49.2	$1.25 \times 1.25 \times 2.0$
Miyazaki et al.^15^	Aortic arch	41.7	$0.885 \times 0.885 \times 1.0$
Soudah et al.^16^	Thoracic aorta	45–49	$1.78 \times 1.78 \times 2.0$

Computational Fluid Dynamics (CFD) is a well known engineering tool used in a wide range of applications which has recently been considered for use in medical applications. It has quickly become an invaluable tool and has been used to achieve multiple advances and breakthroughs^17,18^. A framework was determined to use patient-specific CFD to improve the design of prosthetic heart vales and the implantation procedures^17^, whilst research also used patient-specific CFD to conclude that eccentricity at the aortic root is a major determinant of the haemodynamic patterns in ascending thoracic aortic aneurysm patients^18^ .

By combining 4D-Flow MRI and CFD techniques it is possible to run patient-specific simulations that have the potential to aid treatment planning, disease progression and further the understanding of the haemodynamics due to its predictive capabilities^19–21^. Patient-specific CFD simulations employ geometry and boundary conditions that are determined from 4D-Flow MRI data. Comprehensive methodologies to combine 4D-Flow MRI and CFD in adult patients have been delineated^22,23^. Research has demonstrated the feasibility of combining the two techniques, concluding that the concept has potential to become a useful engineering tool^12^. A number of studies have also shown success in integrating these two techniques^13–16^, however it was noted that the spatial and temporal resolution of the 4D-Flow MRI used to recreate the geometry and inlet conditions was inadequate to fully capture all features of the flow predicted by CFD, specifically the WSS and energy loss^15,16^. The impacts of spatial resolution on the viscous dissipation in patients with fontan circulation was investigated using CFD^24^, however a range of 4D-Flow MRI resolutions was not investigated. Instead, the CFD resolution was altered to match a 4D-Flow MRI resolution. Attempts to minimise the impacts of coarse spatial resolutions of 4D-Flow MRI when using the imaging technique in CFD simulations have been made^25,26^. However, despite the spatial and temporal resolution being a well known limitation to patient-specific CFD simulations, the minimum requirements for spatial resolution when utilising 4D-Flow MRI data in patient-specific CFD simulations have yet to be determined.

In this study the authors investigate the impact 4D-Flow MRI spatial resolution has on subsequent patient-specific CFD simulations with an aim of establishing a minimum spatial resolution requirement needed for producing reliable patient-specific CFD simulations.

## Methods

### Data acquisition

Ethics approval has been given to this study by the Leeds East Research Ethics Committee (18/YH/0439) and Berkshire Research Ethics Committee (10/H0505/100). All data used in this study is anonymous. All participants have given written and informed consent to participate in this study. The study has been performed in accordance with the Declaration of Helsinki. Written and informed consent for publication has been given by all participants in this study.

This study focused on one healthy adult patient with no known heart defect or valve disease. The patient underwent four 4D-Flow MRI scans at 4 different spatial resolutions ($x \times y \times z$ ${\text {mm}}^3$). The four spatial resolutions used were $4\,{\text {mm}} \times 4\,{\text {mm}} \times 4\,{\text {mm}}$, $3\,{\text {mm}} \times 3\,{\text {mm}} \times 3\,{\text {mm}}$, $2\,{\text {mm}} \times 2\,{\text {mm}} \times 2\,{\text {mm}}$, and $1.5\,{\text {mm}} \times 1.5\,{\text {mm}} \times 1.5\,{\text {mm}}$. Scans of the thoracic aorta and proximal supra-aortic vessels were acquired on a 3T Magnetic Resonance system (Siemens 3.0 T PRISMA, Siemens Healthcare, Erlangen, Germany), velocity encoding was set to 150*cm*/*s* in all directions, and flip angle $=7^\circ$. A temporal resolution of $\sim 35$ ms was used for the three coarsest spatial resolutions, and a temporal resolution of $\sim 42$ ms was used for the finest spatial resolution ($1.5\,{\text {mm}} \times 1.5\,{\text {mm}} \times 1.5\,{\text {mm}}$). This was a result of the processing power available of the 4D-Flow MRI scanner used, a temporal resolution of $\sim 35$  ms was not possible with the finest spatial resolution. The $1.5\,{\text {mm}} \times 1.5\,{\text {mm}} \times 1.5\,{\text {mm}}$ scan was approximately 15 min and was of the thoracic aorta only, the $2\,{\text {mm}} \times 2\,{\text {mm}} \times 2\,{\text {mm}}$ was approximately 15 min and was of the whole heart, the $3\,{\text {mm}} \times 3\,{\text {mm}} \times 3\,{\text {mm}}$ was approximately 8 min for the whole heart, and the $4\,{\text {mm}} \times 4\,{\text {mm}} \times 4\,{\text {mm}}$ was approximately 4 min for the whole heart. From each 4D-Flow MRI scan, a geometry was reconstructed and inlet boundary condition extracted. This allowed patient-specific CFD simulations to be run based on each spatial resolution.

### Geometry reconstruction

**Figure 1:**
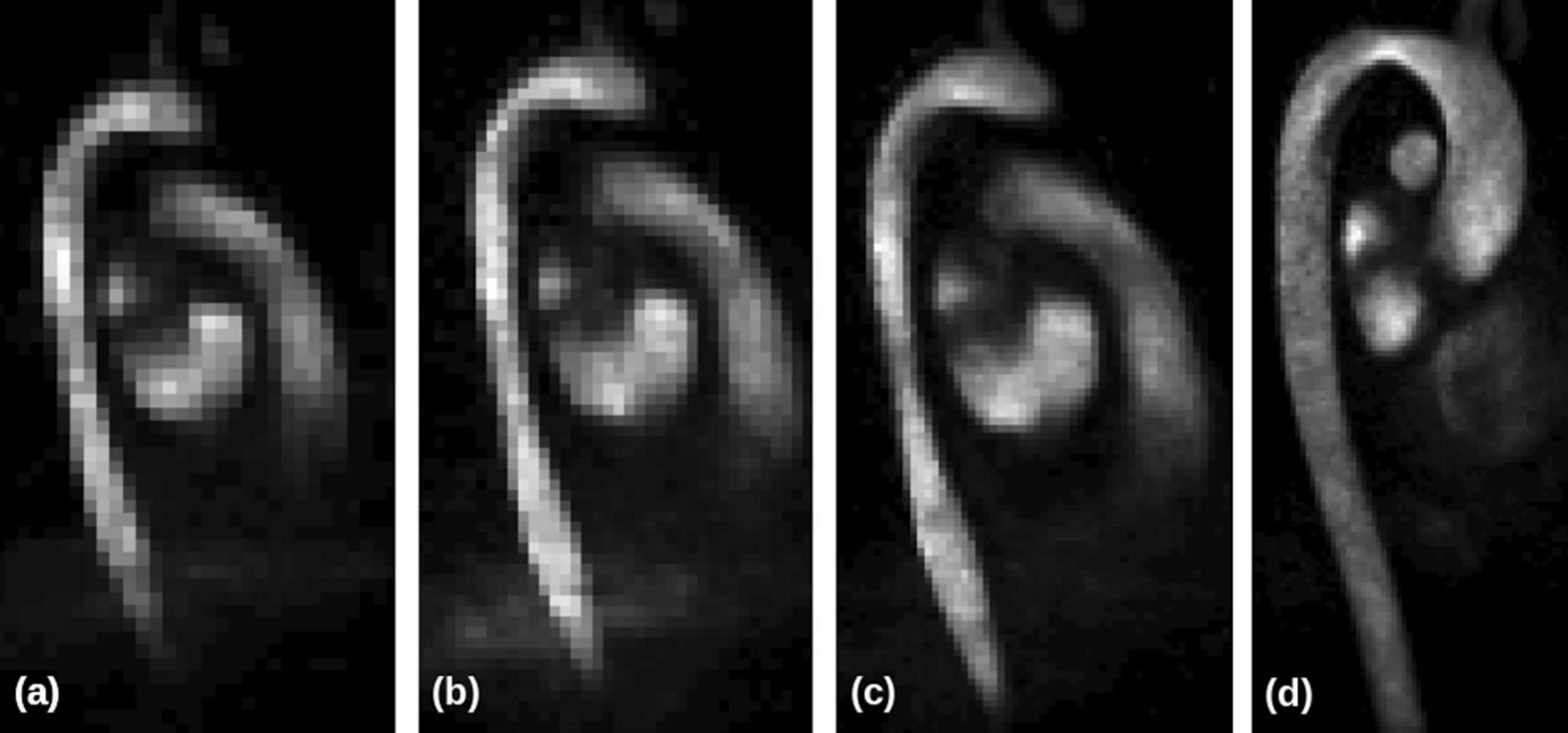
4D-flow MRI images of the thoracic aorta at the same location in the sagittal plane, taken at four varying spatial resolutions ($x \times y \times z$ ${\text {mm}}^3$). (**a**) $4\,{\text {mm}} \times 4\,{\text {mm}} \times 4\,{\text {mm}}$, (**b**) $3\,{\text {mm}} \times 3\,{\text {mm}} \times 3\,{\text {mm}}$, (**c**) $2\,{\text {mm}} \times 2\,{\text {mm}} \times 2\,{\text {mm}}$, (**d**) $1.5\,{\text {mm}} \times 1.5\,{\text {mm}} \times 1.5\,{\text {mm}}$.

**Figure 2:**
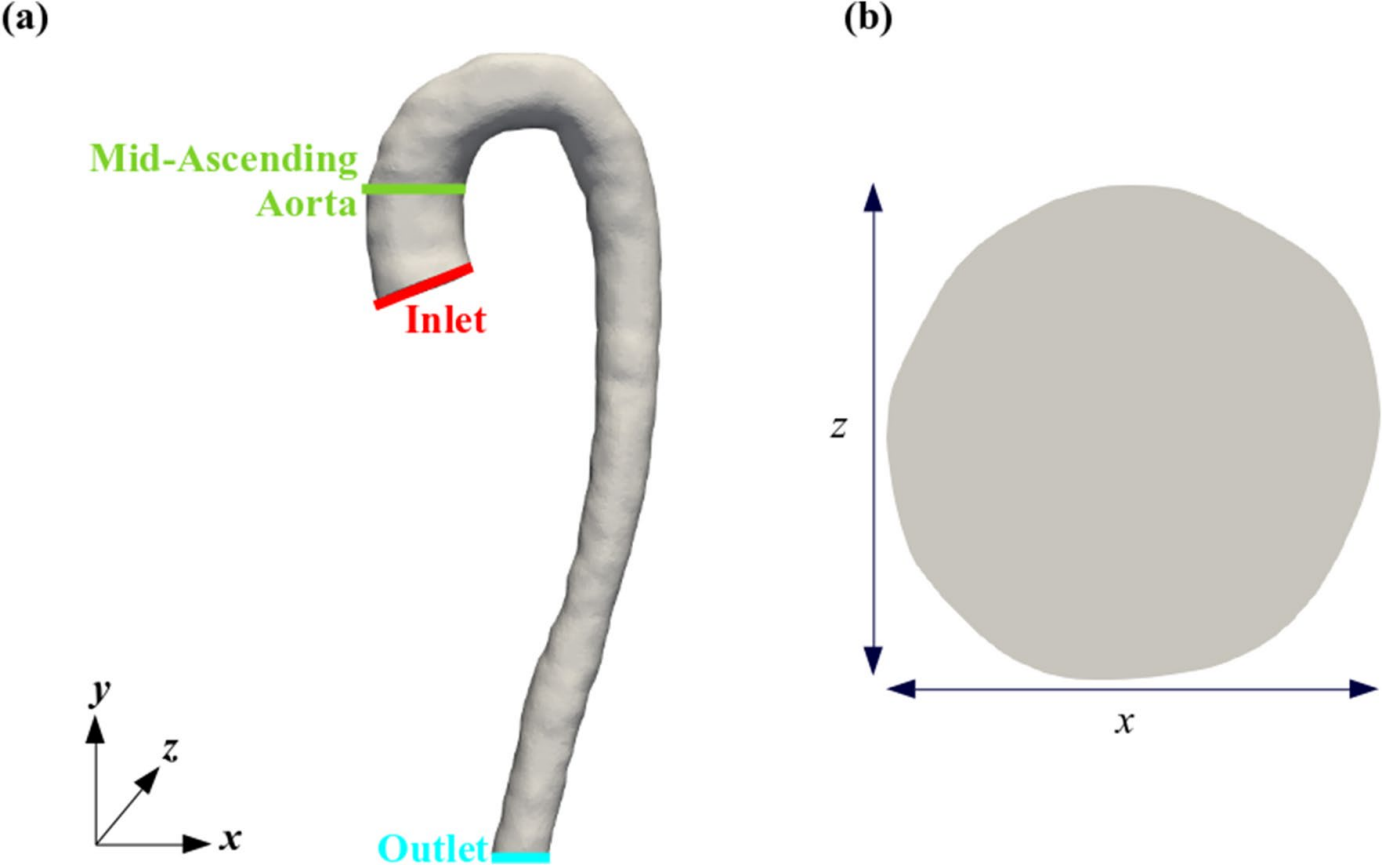
Location of the inlet, outlet, and plane of interest in the mid-ascending aorta for the patient used in this study (**a**), and the cross-section of the aorta in the mid-ascending aortic plane (**b**).

A patient-specific in-silico geometry was reconstructed using images from each 4D-Flow MRI scan. Figure 1 demonstrates the difference in image quality between the spatial resolutions of 4D-Flow MRI scans used. The image quality can be compared quantitatively in Table 2, where the number of voxels used to describe an arbitrary plane in the mid-ascending aorta (diameter $= 25.15 \pm 0.72\,{\text {mm}}$) from each scan resolution is detailed, giving an indication of the accuracy of the spatial resolutions. The location and cross section of the plane used can be seen in Fig. 2.

The proximal thoracic aorta was segmented from all 4D-Flow MRI images in the sagittal, axial, and coronal planes to create an in-silico geometry that can be used in subsequent 4D-Flow MRI and CFD analysis. This geometry reconstruction process was repeated for all spatial resolutions, resulting in four distinct geometries. In all geometries, the supra-aortic vessels were neglected; this was a consequence of low flow velocities present in the vessels meaning they were not fully visible in the 4D-Flow MRI scans combined with the lower resolution scans not providing enough voxels (as recommended^3^) to accurately model the blood flow. The segmentation process within the geometry reconstruction was achieved using in-house code written in MATLAB^27^, similar to that used in other studies^28–30^. The authors will here on refer to the in-house code as the *4D-Flow MRI APP*.

**Figure 3:**
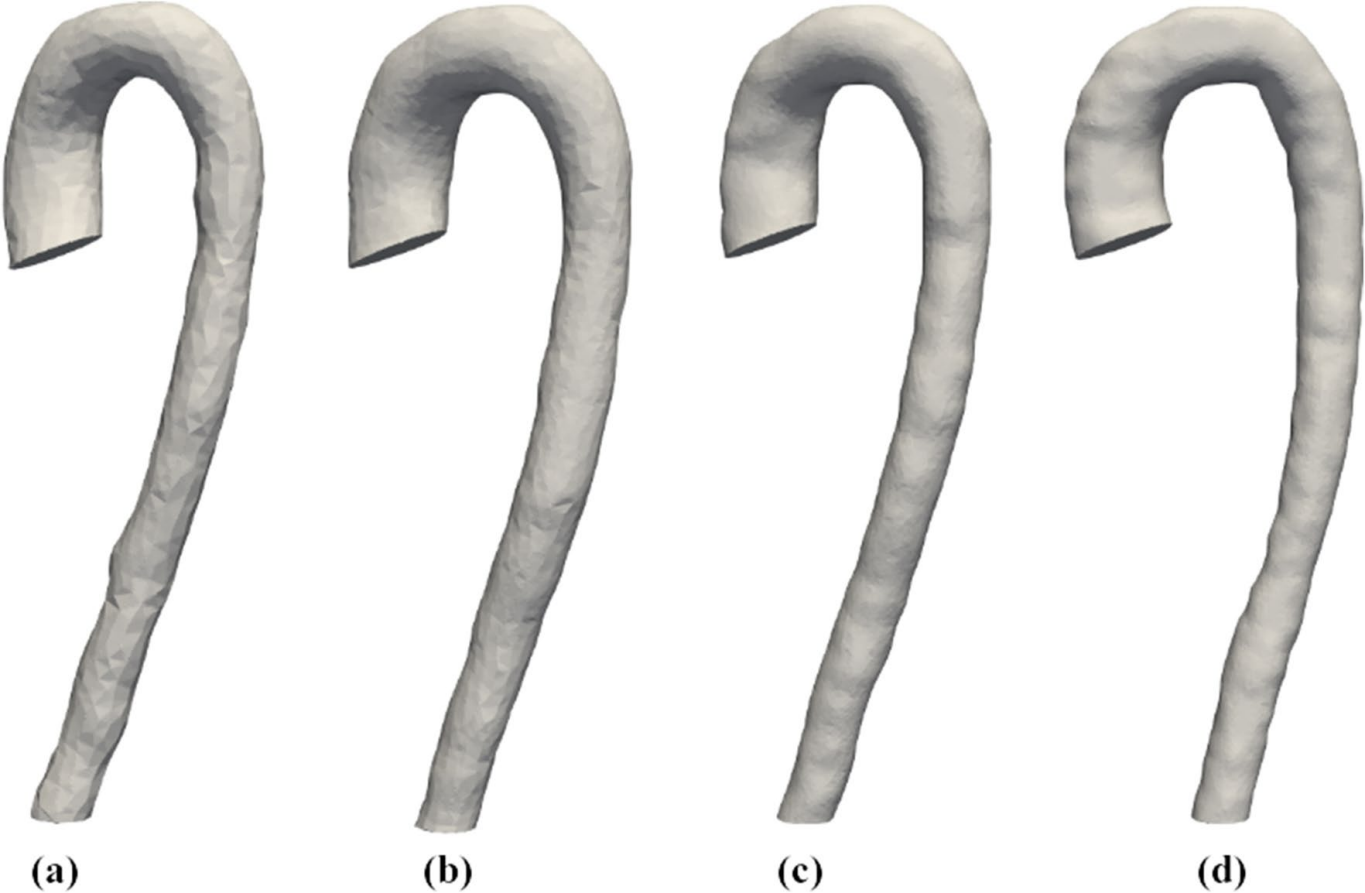
In-silico patient-specific geometries created using 4D-Flow MRI images from the four spatial resolutions using the *4D-Flow MRI APP*, (**a**) $4\,{\text {mm}} \times 4\,{\text {mm}} \times 4\,{\text {mm}}$, (**b**) $3\,{\text {mm}} \times 3\,{\text {mm}} \times 3\,{\text {mm}}$, (**c**) $2\,{\text {mm}} \times 2\,{\text {mm}} \times 2\,{\text {mm}}$, (**d**) $1.5\,{\text {mm}} \times 1.5\,{\text {mm}} \times 1.5\,{\text {mm}}$, visualised in ParaView, 5.3.0^31^.

Using the *4D-Flow MRI APP*, the aortic region was initially segmented out using a threshold, before manually ensuring the correct volume was selected in each image. The four geometries produced using this method are shown in Fig. 3, and are based on each of the corresponding four 4D-Flow MRI spatial resolutions. The reconstructed geometries are based on the maximum dimensions of the thoracic aorta experienced during peak systole. This allows the radial and vertical movement of the aorta throughout the cardiac cycle to be neglected as the reconstructed geometry does not move throughout the cycle. Therefore the plane of interest in the mid-ascending aorta could be selected such that it was in the same local *y* location for each of the 4D-Flow MRI scans ensuring a fair comparison of the haemodynamics in the ascending aorta between the varying spatial resolutions could be made. This also allowed the inlet plane to be placed in the physiologically appropriate location for each geometry reconstruction.

**Table 2:** The number of voxels used in the *x* and *z* directions for the four spatial resolutions to delineate a slice in the axial plane in the mid-ascending aorta.

Scan resolution (mm$^3$)	Number of voxels in *x* direction	Number of voxels in *z* direction
$4 \times 4 \times 4$	7	6
$3 \times 3 \times 3$	10	8
$2 \times 2 \times 2$	15	12
$1.5 \times 1.5 \times 1.5$	20	16

### Meshing

The mesh used for all CFD simulations was created using the *blockMesh* and *snappyHexMesh* utilities within OpenFOAM^32^. A mesh sensitivity study was conducted to ensure all results were independent of the mesh resolution and were impacted only by the spatial resolution of the 4D-Flow MRI scan. The mesh density was increased until it was found that any further increases in mesh produced little change in the velocity magnitude results. Four grids were tested, and the mesh selected for all subsequent simulations was comprised of $\sim 2.3$ million elements, with element sizes in the range of $4.751 \times 10^{-5}m< \delta x < 8.370 \times 10^{-4}m$. The mesh is constructed of structured hexahedra and split hexahedra cells, with refinement regions at the vessel wall. Two refinement levels within the *snappyHexMesh* utility have been used.

### Boundary conditions

The inlet boundary conditions were created from the patient-specific velocity data collected from the 4D-Flow MRI scans. A spatially and temporally varying patient-specific boundary condition was applied to the inlet patch at the inferior end of the ascending aorta (see Fig. 2 for inlet location). The spatio-temporal patient-specific inlet boundary conditions were determined by extracting velocity data at each cell across the inlet location from the 4D-Flow MRI scans. A surface fit was then applied to the extracted data that updated with each time-step to produce the best available approximation of the 4D-Flow MRI data throughout the cardiac cycles. The equation of the surface fit, that varied spatially with the *x* and *z* directions as well as temporally, with *t*, was then applied to the inlet patch of the in-silico model created from the geometry reconstruction process using the *codedFixedBoundary* boundary condition with OpenFOAM. The vessel walls were considered to be non-slip and were assumed to be rigid, and a zero-pressure boundary condition was applied to the outlet at the inferior end of the descending aorta. A zero-pressure boundary condition was selected over the more physiologically accurate 3-element Windkessel model as research suggests that more than 5 diameters upstream of the outlet there is little variation in the flow between a zero-pressure and 3-element Windkessel model^33^. As the plane of interest in this research is more than 5 diameters upstream of the outlet, a zero-pressure boundary condition was deemed an appropriate simplification of the flow.

### CFD simulations

Simulations were run for three cardiac cycles to ensure periodicity was reached using the transient solver *pimpleFoam* for incompressible, turbulent, Newtonian fluids within OpenFOAM^32^. A variable time-step was implemented using the *adjustTimeStep* functionality of *pimpleFoam* with the initial value determined using the Courant Number. All results presented are taken from the last cardiac cycle simulated. As the Reynolds number of the flow varies over the course of the cardiac cycle, the flow moves between the turbulent, transitional, and laminar flow regimes. Because of the turbulent flow present over systole, the k-$\omega$ SST turbulence model was incorporated into the numerical model. The Stokes number at all stages of the cardiac cycle was below the threshold that would suggest the red blood cells, white blood cells, or platelets influence the flow behaviour. Based on this, blood was assumed to be an incompressible, Newtonian, and homogeneous fluid, with a density of $\rho = 1060 kg m^{-3}$ and a dynamic viscosity of $\mu =3.5\times 10^{-3} Pa s$, assuming an average temperature of the human body to be $37^\circ$^34,35^.

All simulations were undertaken on ARC3, part of the high performance computing facilities at the University of Leeds.

## Results

**Figure 4:**
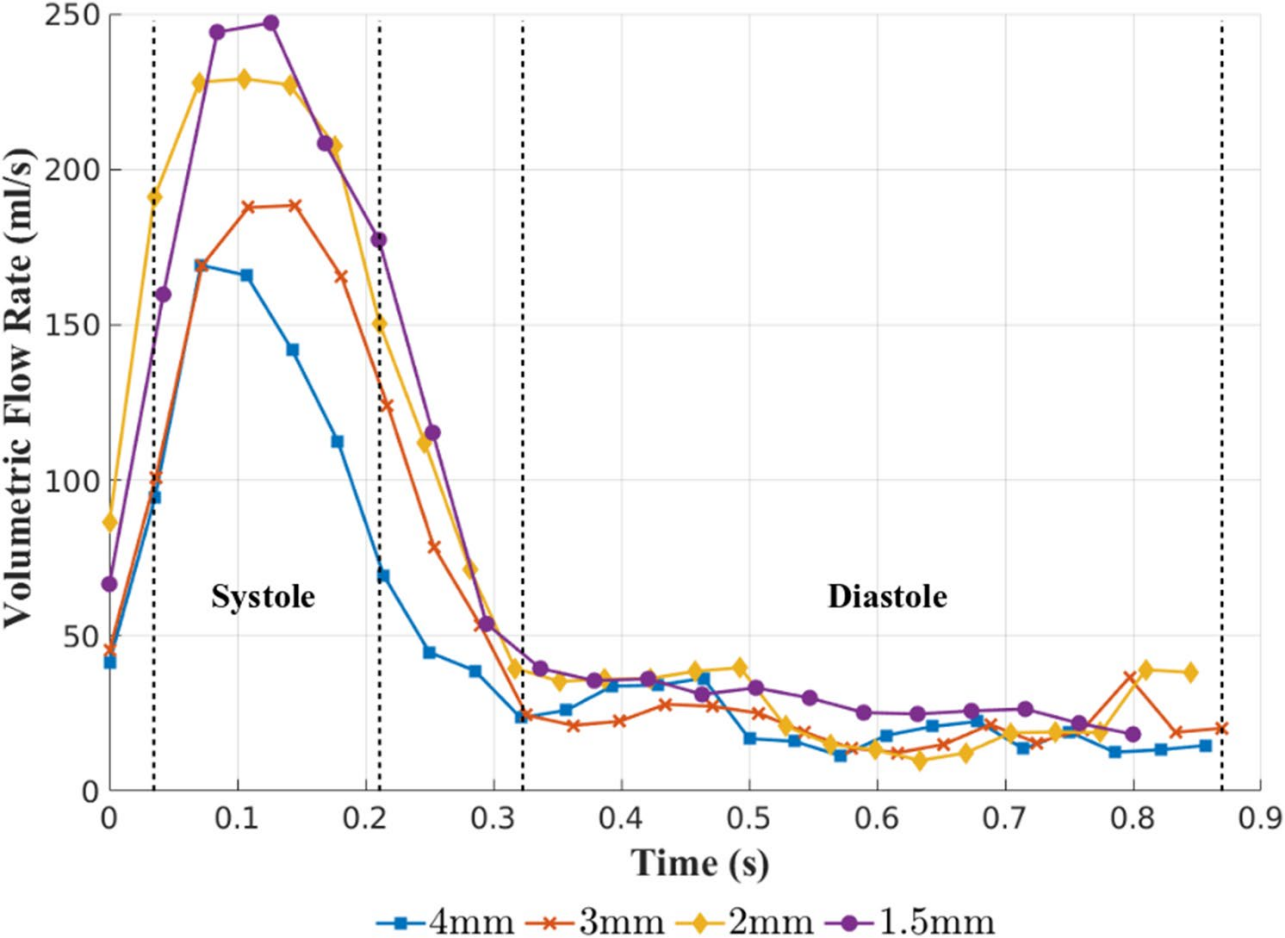
Volumetric flow rates at the inlet determined using 4D-Flow MRI data for all four scan resolutions. The systolic and diastolic phases over which results are averaged are also shown, enclosed by dashed lines.

All results presented in this study have been averaged over the systolic and diastolic phases of the cardiac cycle. The systolic results are determined from the average of the peak systolic time-step, and the time-steps immediately before and after. Diastolic results are determined from averaging all time-steps that are within the diastolic phase. The approximate regions used for the systolic and diastolic results can be seen in Fig. 4. All results are visualised within the open-source software, ParaView^31^.

### Geometry variation

The geometry created from the 4D-Flow MRI data varies between each spatial resolution used. The area of the inlet plane was calculated for each 4D-Flow MRI scan after segmentation had taken place, and for the geometry after meshing used in the subsequent CFD simulations. The diameters of the inlets can be found in Table 3. A clear trend is shown in the data from both 4D-Flow MRI and CFD; as the spatial resolution the 4D-Flow MRI scan is taken at is refined, the diameter of the inlet patch reduces, resulting in the $4\,{\text {mm}} \times 4\,{\text {mm}} \times 4\,{\text {mm}}$ spatial resolution reporting the largest inlet area for both data-sets. The reported diameters for both CFD and 4D-Flow MRI vary noticeably between the four spatial resolutions, with a mean and standard deviation of $2.544cm\pm 0.08346$ and $2.551cm\pm 0.08186$ respectively. However, at each spatial resolution the difference between the diameters reported by CFD and 4D-Flow MRI remains below $1\%$.

### Inlet conditions

The volumetric flow rate at the inlet calculated from 4D-Flow MRI data over the course of the full cardiac cycle can be seen in Fig. 4 for all spatial resolutions. It is clear that as the spatial resolution is refined the volumetric flow rate during systole increases, suggesting a coarse spatial resolution underestimates the volumetric flow rate at the aortic valve. At systole there is a $37.86\%$ difference in the volumetric flow rate at the inlet between the $4\,{\text {mm}} \times 4\,{\text {mm}} \times 4\,{\text {mm}}$ and $1.5\,{\text {mm}} \times 1.5\,{\text {mm}} \times 1.5\,{\text {mm}}$ resolution cases. The differences between the resolutions lessens to $33.03\%$ between $4\,{\text {mm}} \times 4\,{\text {mm}} \times 4\,{\text {mm}}$ and $1.5\,{\text {mm}} \times 1.5\,{\text {mm}} \times 1.5\,{\text {mm}}$ as the cardiac cycle progresses through diastole. This indicates the magnitude of the disparity that is being introduced to patient-specific CFD simulations through the inlet conditions when based on 4D-Flow MRI measurements with inadequate spatial resolution.

**Table 3:** Inlet plane diameters for all 4D-Flow MRI scans calculated using 4D-Flow MRI data and CFD data.

Scan resolution (mm$^3$)	4D-Flow MRI inlet diameter (cm)	CFD inlet diameter (cm)
$4 \times 4 \times 4$	2.659	2.658
$3 \times 3 \times 3$	2.569	2.555
$2 \times 2 \times 2$	2.499	2.491
$1.5 \times 1.5 \times 1.5$	2.478	2.474

**Figure 5:**
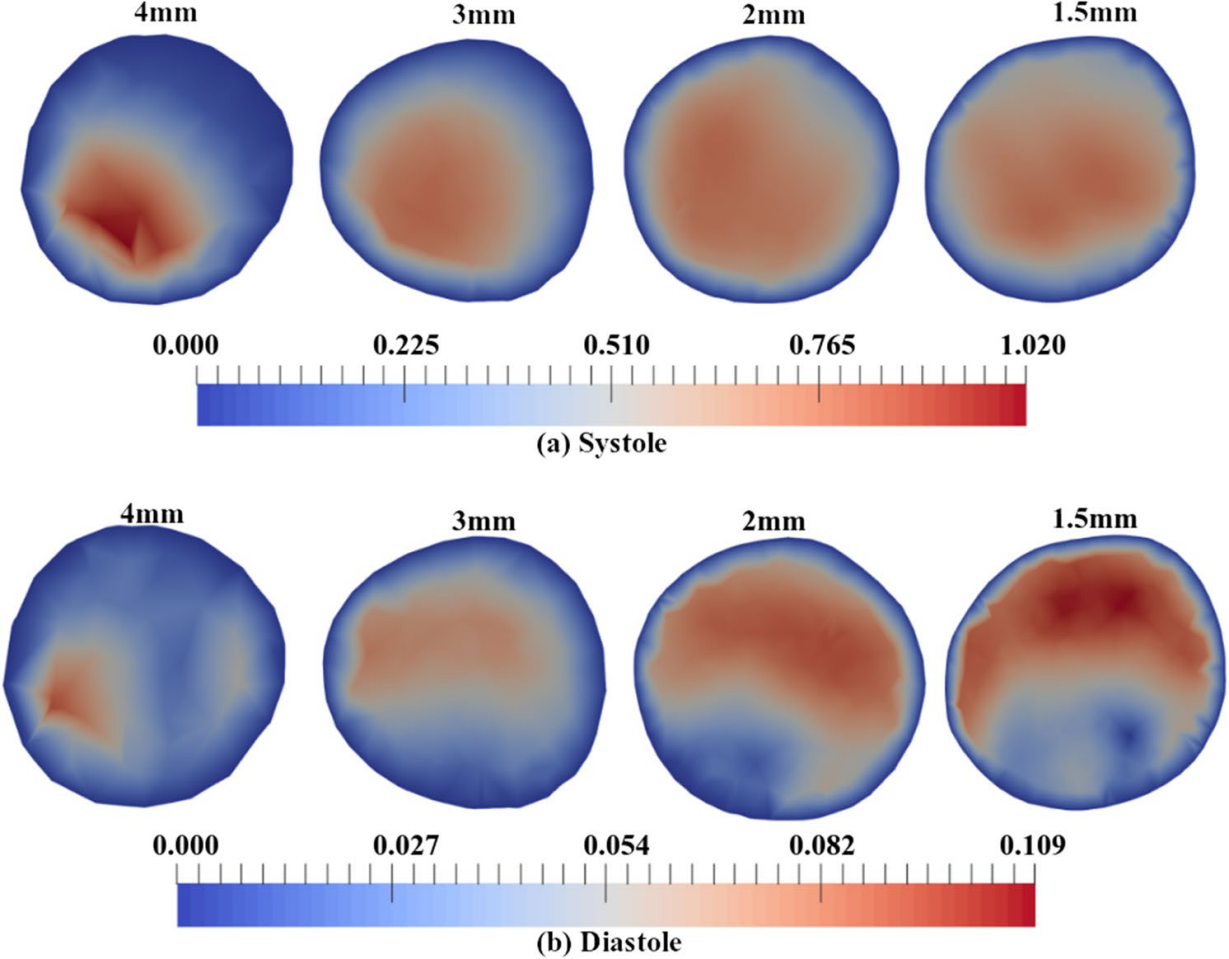
Velocity magnitude (${\text {ms}}^{-1}$) contours at the inlet plane determined from 4D-Flow MRI data for all 4D-Flow MRI spatial resolutions over the systolic and diastolic phases.

The variation introduced to the patient-specific CFD simulations through the inlet conditions is also shown through the velocity magnitude flow patterns at the inlet plane over the systolic and diastolic phases. This can be seen in Fig. 5. When observing data from systole, the magnitude of the maximum velocity appears to be consistent across the $3\,{\text {mm}} \times 3\,{\text {mm}} \times 3\,{\text {mm}}$, $2\,{\text {mm}} \times 2\,{\text {mm}} \times 2\,{\text {mm}}$, and the $1.5\,{\text {mm}} \times 1.5\,{\text {mm}} \times 1.5\,{\text {mm}}$ spatial resolutions (see Fig. 5). However, it is clear that the spatial variation across the inlet plane is considerable between resolutions, notably between the $4\,{\text {mm}} \times 4\,{\text {mm}} \times 4\,{\text {mm}}$ and the finer resolutions. This contributes to the large differences in the volumetric flow rate across the inlet at systole, the magnitudes of which are shown in Fig. 4. The quantitative agreement between the 4D-Flow MRI data at the inlet and the calculated CFD inlet conditions in terms of maximum velocity and mean velocity across the inlet plane can be seen in Table 4 for the systolic phase of the cardiac cycle. It can be seen that as the 4D-Flow MRI spatial resolution is refined, the agreement between the 4D-Flow MRI data and the calculated CFD inlet conditions remains approximately consistent in terms of the maximum velocity experienced: within $<2\%$ of each other regardless of the spatial resolution the data is acquired at, suggesting the inlet conditions are accurately capturing the 4D-Flow MRI data. As the spatial resolution is refined, the agreement between two methods increases when observing the mean velocity over the inlet patch; a spatial resolution of $4\,{\text {mm}} \times 4\,{\text {mm}} \times 4\,{\text {mm}}$ produces a $23.37\%$ difference in the mean velocity, reducing to a $8.192\%$ difference with a spatial resolution of $1.5\,{\text {mm}} \times 1.5\,{\text {mm}} \times 1.5\,{\text {mm}}$. It can also be seen in Table 4 that the trends present in the 4D-Flow MRI data across the range of resolutions are also present in the CFD data.

From Fig. 5, it can be seen that during the diastolic phase of the cardiac cycle the flow patterns from 4D-Flow MRI data are in general qualitative agreement for spatial resolutions of $3\,{\text {mm}} \times 3\,{\text {mm}} \times 3\,{\text {mm}}$, $2\,{\text {mm}} \times 2\,{\text {mm}} \times 2\,{\text {mm}}$, and $1.5\,{\text {mm}} \times 1.5\,{\text {mm}} \times 1.5\,{\text {mm}}$. As the spatial resolution of 4D-Flow MRI data increases, so too does the maximum velocity experienced at the inlet, as well as the mean velocity across the inlet patch. This trend is echoed, as expected, in the inlet conditions of the subsequent patient-specific CFD simulations. As the 4D-Flow MRI spatial resolution is refined, the agreement between 4D-Flow MRI and CFD data in terms of the maximum velocity experienced and the mean velocity over the inlet patch increases, as seen in Table 5. The agreement in maximum velocity values increases from being within $14.91\%$ to $3.613\%$ of each other, whilst the mean velocity agreement increases from the values being within $22.70\%$ to $6.285\%$ of each other. Similarly to the data at systole, it can be seen that there is considerable variation in the maximum and mean velocity between the spatial resolutions for both 4D-Flow MRI data and the calculated CFD inlet conditions.

It is evident that during both systole and diastole, the $4\,{\text {mm}} \times 4\,{\text {mm}} \times 4\,{\text {mm}}$ resolution data disagrees both in terms of flow patterns and velocity magnitude when compared to all other spatial resolutions. The maximum velocity over the inlet plane during systole varies considerably between the various resolutions when looking at 4D-Flow MRI data ($0.8554 \pm 0.1085\,{\text {ms}}^{-1}$). This variation is translated into the subsequent CFD inlet conditions ($0.8574 \pm 0.09462\,{\text {ms}}^{-1}$). During the diastolic phase, the variation between the maximum velocity between the various resolutions is slightly reduced, with the 4D-Flow MRI data reporting $0.1009 \pm 0.006474 \,{\text {ms}}^{-1}$, and CFD reporting $0.09988 \pm 0.009979\, {\text {ms}}^{-1}$.

**Table 4:** Velocity magnitude (${\text {ms}}^{-1}$) data at the inlet plane during systole calculated using 4D-Flow MRI data and CFD data, including a comparison between the two methods for each spatial resolution.

	4D-Flow MRI		CFD		Difference between methods	
	${U_{mag}}_{max}$ (${\text {ms}}^{-1}$)	$U_{mean}$ (${\text {ms}}^{-1}$)	${U_{mag}}_{max}$ (${\text {ms}}^{-1}$)	$U_{mean}$ (${\text {ms}}^{-1}$)	${U_{mag}}_{max}$ $\%$ difference	$U_{mean}$ $\%$ difference
4 mm	1.018	0.2838	0.9988	0.3589	1.904	23.37
3 mm	0.8052	0.3745	0.8209	0.4511	1.931	18.56
2 mm	0.7986	0.4683	0.8010	0.5333	0.3001	12.98
1.5 mm	0.7997	0.4835	0.8089	0.5248	1.144	8.192

**Table 5:** Velocity magnitude (${\text {ms}}^{-1}$) data at the inlet plane during diastole calculated using 4D-Flow MRI data and CFD data, including a comparison between the two methods for each spatial resolution.

	4D-Flow MRI		CFD		Difference between methods	
	${U_{mag}}_{max}$ (${\text {ms}}^{-1}$)	$U_{mean}$ (${\text {ms}}^{-1}$)	${U_{mag}}_{max}$ (${\text {ms}}^{-1}$)	$U_{mean}$ (${\text {ms}}^{-1}$)	${U_{mag}}_{max}$ $\%$ difference	$U_{mean}$ $\%$ difference
4 mm	0.1038	0.03710	0.08940	0.04660	14.91	22.70
3 mm	0.09520	0.04420	0.09540	0.05480	0.2099	21.41
2 mm	0.09600	0.05520	0.1020	0.06300	6.061	13.20
1.5 mm	0.1087	0.06010	0.1127	0.06400	3.613	6.285

### Velocity

**Figure 6:**
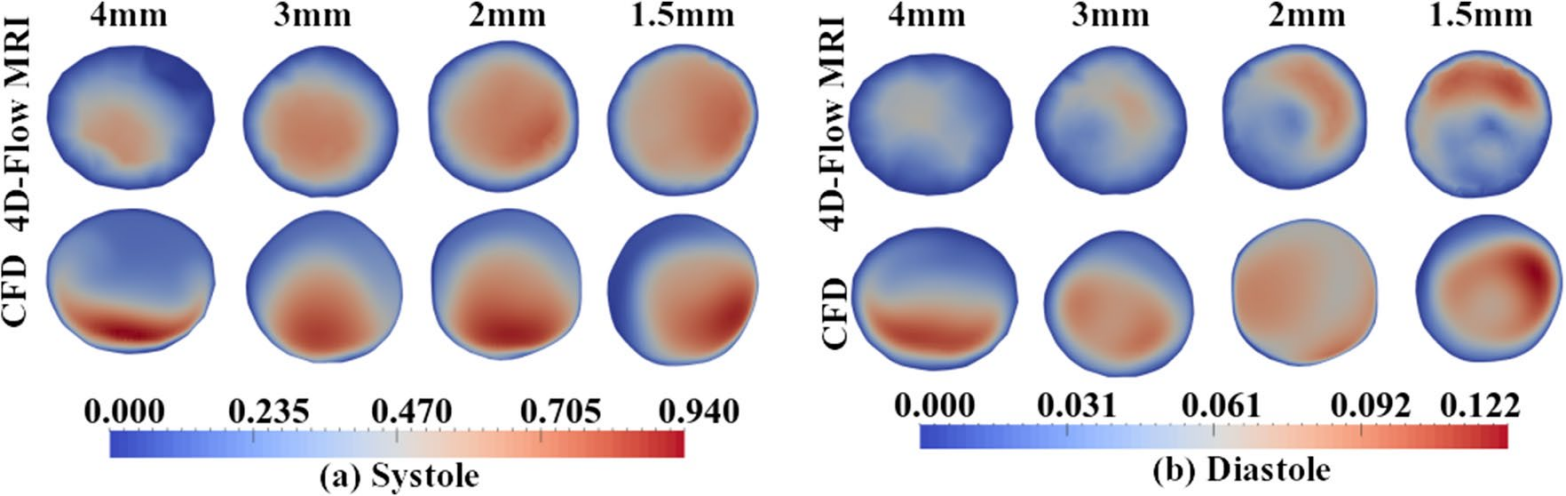
Averaged systolic and diastolic velocity magnitude $({\text {ms}}^{-1})$ contours over an axial slice in the mid ascending aorta, comparing 4D-Flow MRI and CFD for all spatial resolutions.

Contours of velocity magnitude at an axial plane in the mid-ascending aorta during systole can be seen in Fig. 6. Results from patient-specific CFD simulations are compared to the corresponding 4D-Flow MRI data for all spatial resolutions. Figure 6 demonstrates that as the spatial resolution the 4D-Flow MRI data is acquired at is refined, the maximum velocity magnitude experienced across the plane of interest increases for both 4D-Flow MRI data and CFD data, with the notable exception of the patient-specific CFD simulation based on the $4\,{\text {mm}} \times 4\,{\text {mm}} \times 4\,{\text {mm}}$ spatial resolution. The same trends seen over systole (Fig. 6) are also present in the velocity magnitude contours during diastole (Fig. 6). A coarser spatial resolution appears to predict a lower value for the maximum velocity magnitude experienced across the plane of interest, with the exception of the patient-specific CFD simulation based on the $4\,{\text {mm}} \times 4\,{\text {mm}} \times 4\,{\text {mm}}$ spatial resolution.

**Figure 7:**
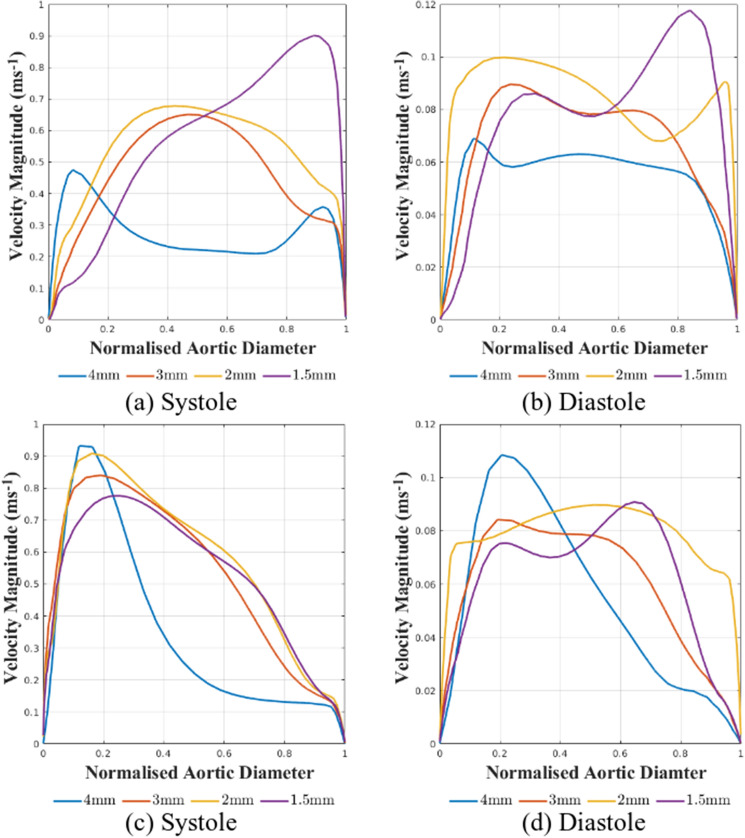
Velocity magnitude along the x-axis (**a**,**b**) and z-axis (**c**,**d**) across a slice in the mid-ascending aorta during the systolic and diastolic phases for all CFD simulations based on the four spatial resolutions of 4D-Flow MRI scan.

At systole, it can be seen that a $4\,{\text {mm}} \times 4\,{\text {mm}} \times 4\,{\text {mm}}$ spatial resolution demonstrates notable discrepancies between the 4D-Flow MRI data and the patient-specific CFD simulation, with much higher velocity magnitudes and differing flow patterns being predicted in the patient-specific CFD simulations, resulting in a maximum velocity magnitude that differs between CFD data and 4D-Flow MRI data by $39.49\%$, and the mean velocity differing by $24.85\%$. The $3\,{\text {mm}} \times 3\,{\text {mm}} \times 3\,{\text {mm}}$, $2\,{\text {mm}} \times 2\,{\text {mm}} \times 2\,{\text {mm}}$, and $1.5\,{\text {mm}} \times 1.5\,{\text {mm}} \times 1.5\,{\text {mm}}$ spatial resolutions demonstrate an increased agreement between the CFD results and 4D-Flow MRI data both in terms of velocity magnitude and flow patterns present compared to the $4\,{\text {mm}} \times 4\,{\text {mm}} \times 4\,{\text {mm}}$ spatial resolution, with differences in the maximum velocity magnitude between CFD and 4D-Flow MRI reducing to $19.44\%$, $14.19\%$, and $16.31\%$ for the $3\,{\text {mm}} \times 3\,{\text {mm}} \times 3\,{\text {mm}}$, $2\,{\text {mm}} \times 2\,{\text {mm}} \times 2\,{\text {mm}}$, and $1.5\,{\text {mm}} \times 1.5\,{\text {mm}} \times 1.5\,{\text {mm}}$respectively. CFD data over-estimates the velocity magnitude compared to the 4D-Flow MRI data at all spatial resolutions. The mean velocity difference between the 4D-Flow MRI data and CFD data reduces to $1.476\%$ for the $3\,{\text {mm}} \times 3\,{\text {mm}} \times 3\,{\text {mm}}$ and $2\,{\text {mm}} \times 2\,{\text {mm}} \times 2\,{\text {mm}}$ spatial resolution cases, whilst the $1.5\,{\text {mm}} \times 1.5\,{\text {mm}} \times 1.5\,{\text {mm}}$ produces a difference in mean velocity of $13.49\%$ between the two methods. This data is summarised within Table 6. The increasing difference seen in the $1.5\,{\text {mm}} \times 1.5\,{\text {mm}} \times 1.5\,{\text {mm}}$ resolution compared to the $2\,{\text {mm}} \times 2\,{\text {mm}} \times 2\,{\text {mm}}$ resolution is likely a product of the coarser temporal resolution used to acquire the finest spatial resolution. This increasing difference in mean velocity can be seen in Fig. 7, where the velocity profile in the *x*-axis is plotted. It can be seen that the $3\,{\text {mm}} \times 3\,{\text {mm}} \times 3\,{\text {mm}}$ and $2\,{\text {mm}} \times 2\,{\text {mm}} \times 2\,{\text {mm}}$ cases demonstrate similar profiles with peak values close in range whilst the $1.5\,{\text {mm}} \times 1.5\,{\text {mm}} \times 1.5\,{\text {mm}}$ displays a alternate flow profile with a higher peak value. The velocity profile along the *z*-axis can also be seen in Fig. 7. It can be seen that at systole, the $4\,{\text {mm}} \times 4\,{\text {mm}} \times 4\,{\text {mm}}$ produces an alternate profile to the remaining spatial resolutions, which all display similar velocity profiles with small variations in magnitude. It can be seen that the $1.5\,{\text {mm}} \times 1.5\,{\text {mm}} \times 1.5\,{\text {mm}}$ demonstrates a lower velocity magnitude than the $2\,{\text {mm}} \times 2\,{\text {mm}} \times 2\,{\text {mm}}$. This is likely a result of the flow patterns between the resolutions altering; Fig. 6 suggests the peak velocity location shifts from the inferior region towards the right-inferior region at the highest spatial resolution.

**Table 6:** Velocity magnitude (${\text {ms}}^{-1}$) data at the mid-ascending aortic plane during systole calculated using 4D-Flow MRI data and CFD data, including a comparison between the two methods for each spatial resolution.

	4D-Flow MRI		CFD		Difference between methods	
	${U_{mag}}_{max}$ (${\text {ms}}^{-1}$)	$U_{mean}$ (${\text {ms}}^{-1}$)	${U_{mag}}_{max}$ (${\text {ms}}^{-1}$)	$U_{mean}$ (${\text {ms}}^{-1}$)	${U_{mag}}_{max}$ $\%$ difference	$U_{mean}$ $\%$ difference
4 mm	0.6272	0.2646	0.9358	0.3396	39.49	24.85
3 mm	0.6941	0.3958	0.8436	0.4016	19.44	1.476
2 mm	0.7891	0.4707	0.9096	0.4777	14.19	1.476
1.5 mm	0.7659	0.4910	0.9019	0.4289	16.31	13.49

In agreement with results at systole, it can be seen that at diastole the CFD data based on the $4\,{\text {mm}} \times 4\,{\text {mm}} \times 4\,{\text {mm}}$ spatial resolution appears to over-estimate the velocity magnitude as well as producing incorrect flow patterns, when compared to the 4D-Flow MRI data of the same spatial resolution, with a difference in the maximum velocity of $56.76\%$, and $60.59\%$ in the mean velocity between the CFD and 4D-Flow MRI data. However, the agreement between 4D-Flow MRI data and the patient-specific CFD simulations increases as the spatial resolution of the 4D-Flow MRI is refined. The $3\,{\text {mm}} \times 3\,{\text {mm}} \times 3\,{\text {mm}}$ reports a difference in maximum velocity of $23.53\%$ between the methods, $2\,{\text {mm}} \times 2\,{\text {mm}} \times 2\,{\text {mm}}$ suggests a $4.471\%$ difference, which is further reduced to $3.670\%$ for a spatial resolution of $1.5\,{\text {mm}} \times 1.5\,{\text {mm}} \times 1.5\,{\text {mm}}$. This data is summarised within Table 7.

It can be seen in Fig. 6 that at diastole there are discrepancies in the flow patterns present across the plane of interest, not only when comparing 4D-Flow MRI results to the corresponding CFD data of the same spatial resolution, but also when comparing the various spatial resolutions to each other for both 4D-Flow MRI data and CFD data. These differences in flow patterns result in CFD simulations over-estimating the mean velocity over the plane of interest considerably for all spatial resolutions. This is further shown by Fig. 7, which demonstrates the various flow profiles across the *x*-axis over the diastolic phase of the cardiac cycle determined from CFD data. It can be seen that a different flow profile is present for each spatial resolution, with the $1.5\,{\text {mm}} \times 1.5\,{\text {mm}} \times 1.5\,{\text {mm}}$ spatial resolution displaying a higher peak value than the remaining spatial resolutions. This is likely a result of the coarser temporal resolution that was utilised when acquiring the data. It is also possible that the inter-scan variability of the patients heart rate could be contributing the differences between the scans. The velocity magnitude plotted along the *z*-axis also demonstrates all four resolutions give a range in flow profiles, similarly to results at systole, the $4\,{\text {mm}} \times 4\,{\text {mm}} \times 4\,{\text {mm}}$ demonstrates the highest peak velocity magnitude.

When comparing the variability of the CFD results between spatial resolutions, it can be seen that there is variation in the results at both systole and diastole. During the systolic phase of the cardiac cycle, over all spatial resolutions investigated, the average velocity and the standard deviation for the CFD data is calculated to be $0.4120\pm 0.05758\,{\text {ms}}^{-1}$, whilst the corresponding value for 4D-Flow MRI data is calculated to be $0.4055\pm 0.1025\,{\text {ms}}^{-1}$. During the diastolic phase of the cardiac cycle, there is a $21.42\%$ difference in the average velocity over the plane of interest between the CFD results based on the $4\,{\text {mm}} \times 4\,{\text {mm}} \times 4\,{\text {mm}}$ and the $1.5\,{\text {mm}} \times 1.5\,{\text {mm}} \times 1.5\,{\text {mm}}$ spatial resolutions. Over all four spatial resolutions, the average velocity over the plane of interest and the standard deviation was calculated to be $0.05286\pm 0.005113 \,{\text {ms}}^{-1}$, whilst the corresponding value for 4D-Flow MRI data is $0.03316\pm 0.006809\, {\text {ms}}^{-1}$. This demonstrates that there is more variability within the 4D-Flow MRI data than the CFD data during the systolic and diastolic phases of the cardiac cycle.

Figure 7 shows the velocity magnitude calculated through OpenFOAM simulations along the x-axis of a plane in the mid-ascending aorta during systole and diastole. At both stages of the cardiac cycle it is clear that results based on the $1.5\,{\text {mm}} \times 1.5\,{\text {mm}} \times 1.5\,{\text {mm}}$present a different flow profile, and at peak systole appears to predict a much higher peak magnitude than the CFD simulations based on a coarser 4D-Flow MRI spatial resolution. This difference can be attributed to the coarser temporal resolution that is used for the $1.5\,{\text {mm}} \times 1.5\,{\text {mm}} \times 1.5\,{\text {mm}}$4D-Flow MRI. It can also be seen from Fig. 7 that the velocities from all spatial resolutions are not yet converging on a velocity magnitude or flow pattern at either stage of the cardiac cycle. As there is no true ’gold-standard’ to compare the results to it must be assumed that results based on the $1.5\,{\text {mm}} \times 1.5\,{\text {mm}} \times 1.5\,{\text {mm}}$ spatial resolution are also being affected by the spatial and temporal resolution of the 4D-Flow MRI scan. As the temporal resolution for this scan is different to the other scans it is not possible to decipher which has the biggest influence over the results.

**Table 7:** Velocity magnitude (${\text {ms}}^{-1}$) data at the mid-ascending aortic plane during diastole calculated using 4D-Flow MRI data and CFD data, including a comparison between the two methods for each spatial resolution.

	4D-Flow MRI		CFD		Difference between methods	
	${U_{mag}}_{max}$ (${\text {ms}}^{-1}$)	$U_{mean}$ (${\text {ms}}^{-1}$)	${U_{mag}}_{max}$ (${\text {ms}}^{-1}$)	$U_{mean}$ (${\text {ms}}^{-1}$)	${U_{mag}}_{max}$ $\%$ difference	$U_{mean}$ $\%$ difference
4 mm	0.06131	0.02599	0.1099	0.04859	56.76	60.59
3 mm	0.07451	0.02902	0.09438	0.05193	23.53	56.61
2 mm	0.09052	0.03700	0.09466	0.05067	4.471	31.18
1.5 mm	0.1177	0.04063	0.1221	0.06024	3.670	38.88

### Wall shear stress

**Figure 8:**
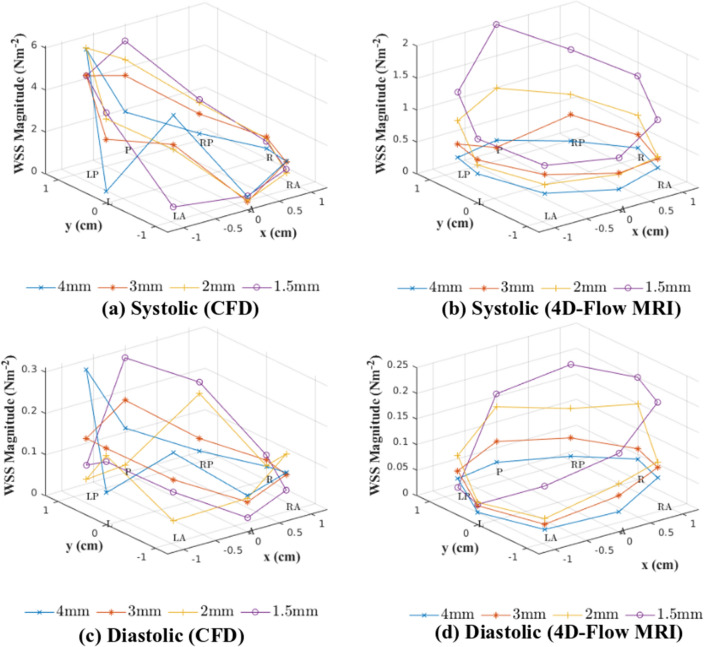
Wall shear stress magnitude values at eight locations on the thoracic aorta wall at a plane in the mid ascending aorta determined through 4D-Flow MRI and CFD for all four resolutions over the systolic (**a**,**b**) and diastolic (**c**,**d**) phase of the cardiac cycle. *R* right, *RP* right-posterior, *P* posterior, *LP* left-posterior, *L* left, *LA* left-anterior, *A* anterior, *RA* right-anterior.

The WSS was calculated from CFD and 4D-Flow MRI data at eight locations on the vessel wall at a slice in the mid-ascending aorta during systole and diastole. From this the variation in the WSS measurements due to the spatial resolution of the 4D-Flow MRI scan can be quantified. Plots of WSS magnitude can be seen in Fig. 8 for systole and diastole. When looking at the WSS plots calculated directly from 4D-Flow MRI data, it can be seen that a refinement in the spatial resolution of the 4D-Flow MRI scan produces an increase in the WSS magnitude experienced at both stages of the cardiac cycle. The $1.5\,{\text {mm}} \times 1.5\,{\text {mm}} \times 1.5\,{\text {mm}}$ spatial resolution scan produces the highest WSS magnitude at both systole and diastole when compared to other spatial resolutions. The WSS results determined from patient-specific CFD simulations do not appear to follow the same trend, and all four sets of results appear to predict WSS values of similar magnitudes.

During systole, results from all CFD simulations indicate a region of elevated WSS in the posterior and left-posterior region. This is also indicated in results from the 4D-Flow MRI data from the $1.5\,{\text {mm}} \times 1.5\,{\text {mm}} \times 1.5\,{\text {mm}}$ spatial resolution. It is not present in 4D-Flow MRI data for spatial resolutions coarser than the $1.5\,{\text {mm}} \times 1.5\,{\text {mm}} \times 1.5\,{\text {mm}}$ resolution. However, the data from CFD simulations predicts elevated levels of WSS when compared to the 4D-Flow MRI data in the posterior and left-posterior region. In all other regions, the magnitude of the WSS predicted through CFD simulations matches the results from 4D-Flow MRI to a much better degree. Statistical comparison (Wilcoxon signed rank test, $\alpha =0.05$) between the CFD data and 4D-Flow MRI data at systole determined the differences found for the $4\,{\text {mm}} \times 4\,{\text {mm}} \times 4\,{\text {mm}}$, $3\,{\text {mm}} \times 3\,{\text {mm}} \times 3\,{\text {mm}}$, and $2\,{\text {mm}} \times 2\,{\text {mm}} \times 2\,{\text {mm}}$ spatial resolutions are all statistically significant ($p<0.05$), whilst comparison between the CFD and 4D-Flow MRI data for the $1.5\,{\text {mm}} \times 1.5\,{\text {mm}} \times 1.5\,{\text {mm}}$ spatial resolution concluded the differences were insignificant. This suggests a coarse spatial resolution has notable impacts on the accuracy of WSS measurements in any subsequent CFD simulations that are performed, and a spatial resolution of $1.5\,{\text {mm}} \times 1.5\,{\text {mm}} \times 1.5\,{\text {mm}}$ provides reasonable agreement between 4D-Flow MRI data and results from patient-specific CFD simulations.

During diastole, the CFD data does not follow the trend of an increasing spatial resolution producing higher levels of WSS that is seen in 4D-Flow MRI data. Figure 8 indicates that CFD simulations predict WSS magnitudes of similar size regardless of the spatial resolution of the 4D-Flow MRI data they are based on. However, as the spatial resolution is refined, the agreement between the CFD data and the 4D-Flow MRI data improves, with the differences between the $4\,{\text {mm}} \times 4\,{\text {mm}} \times 4\,{\text {mm}}$ being statistically significant ($p<0.05$) and the results for all other spatial resolutions being insignificant.

**Figure 9:**
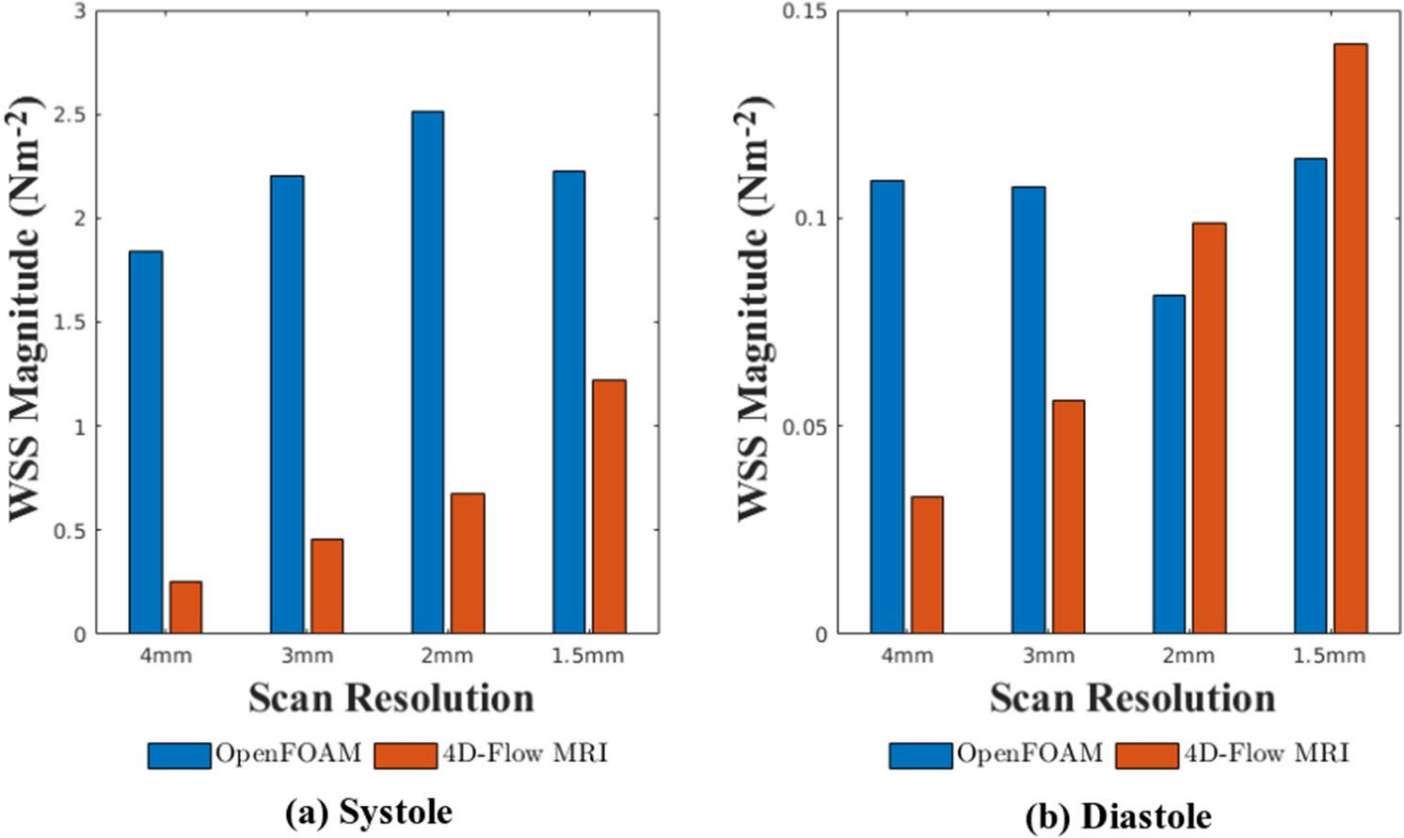
Average wall shear stress magnitude over eight locations of the vessel wall for a plane in the mid-ascending aorta, calculated with 4D-Flow MRI and CFD data for all spatial resolutions during systole and diastole.

Figure 9 demonstrates the average WSS value at the vessel wall across the plane of interest. During systole and diastole, it is clear that as the spatial resolution is refined, the 4D-Flow MRI data shows increasing WSS magnitudes. This leads to improved levels of agreement between the 4D-Flow MRI data and the results from patient-specific CFD simulations as the spatial resolution is refined. When comparing the agreement levels between the CFD data and the 4D-Flow MRI data for the $4\,{\text {mm}} \times 4\,{\text {mm}} \times 4\,{\text {mm}}$ at systole, there is a $151.9\%$ difference, which reduces to $58.09\%$ as the spatial resolution is increased to $1.5\,{\text {mm}} \times 1.5\,{\text {mm}} \times 1.5\,{\text {mm}}$. This is mirrored at diastole, with the difference between methods being $106.7\%$ for the $4\,{\text {mm}} \times 4\,{\text {mm}} \times 4\,{\text {mm}}$ spatial resolution reducing to a difference of $21.67\%$ for the $1.5\,{\text {mm}} \times 1.5\,{\text {mm}} \times 1.5\,{\text {mm}}$ spatial resolution. When observing the mean and standard deviation of the WSS results, it can be seen that there is much greater variability across all spatial resolutions in the WSS measurements taken from 4D-Flow MRI data at both systole ($0.6509 \pm 0.4184\,{\text {Nm}}^{-2}$) and diastole ($0.08250 \pm 0.04795 \,{\text {Nm}}^{-2}$), compared to CFD results at systole ($2.1960 \pm 0.2767 \,{\text {Nm}}^{-2}$) and diastole ($0.1030 \pm 0.01469 \,{\text {Nm}}^{-2}$).

## Discussion

The diameters of the inlet patch vary slightly between the four spatial resolutions tested within this study, whilst there is excellent agreement between the 4D-Flow MRI data and CFD data at each spatial resolution. It is clear that as the spatial resolution is refined, the area of the inlet patch decreases. This highlights the relationship between the vessel diameter and the resolution: the diameter of the vessel is sensitive to the spatial resolution used to acquire the data. As this is occurring at the inlet plane, it can be assumed that the same trend will be prevalent at all locations within the thoracic aorta. From this, it can be expected that a consequence of acquiring 4D-Flow MRI data using an insufficient spatial resolution is a sizeable overestimation of the size of the vessel. It must also be noted that the segmentation and thresholding of the 4D-Flow MRI data undertaken during the geometry reconstruction process may also be contributing to the overestimation of the vessel diameter. However, it is unknown to what degree a systematic error contributes to the overestimation as there is no ’gold standard’ available to compare the geometry to. Despite the possibility of a systematic error being introduced, as the same method was applied to all four geometry reconstructions, the presence of discrepancies in diameter between the spatial resolutions strongly indicates that it is the spatial resolution the 4D-Flow MRI is acquired at that is the main cause of the range in diameters.

There is variation in the inlet conditions that are found from 4D-Flow MRI data when comparing all four spatial resolutions at both systole and diastole, with the coarser spatial resolutions displaying a more sharp profile, and the finer spatial resolutions produced a broader flow profile. The spatial variations present between the four resolutions combined with the range in inlet areas produces considerable differences in the volumetric flow rates of all four spatial resolutions. The discrepancies at the inlet between the 4D-Flow MRI and CFD data are most notable for the $4\,{\text {mm}} \times 4\,{\text {mm}} \times 4\,{\text {mm}}$ spatial resolution, where the agreement between the methods is poor, both in terms of the flow patterns observed, but also the magnitude of the velocity. It must be acknowledged that the natural variations within the patients beat may also be contributing to small differences between the four 4D-Flow MRI acquisitions.

As the spatial resolution is increased, the agreement between the 4D-Flow MRI data and the CFD inlet conditions increases. This behaviour is expected due to the method used to calculate the spatio-temporal patient-specific inlet conditions for the CFD simulations: a fit is calculated from the 4D-Flow MRI data and is subsequently applied to the CFD inlet patch. A coarse 4D-Flow MRI spatial resolution has fewer data-points across the inlet plane to extract velocity data from that can be used to create a surface fit from. This results is a lesser quality of fit as a higher degree of interpolation is required between the 4D-Flow MRI data points, therefore a higher interpolation error is expected in the calculated inlet conditions with lower 4D-Flow MRI spatial resolutions. As the number of data-points across the inlet location that velocity data is extracted from is increased (as the spatial resolution is increased), the quality of the surface fit is improved as less interpolation is required between the 4D-Flow MRI data points, resulting in improved agreement between the 4D-Flow MRI data and the CFD inlet conditions. However, as the 4D-Flow MRI velocity data is not mapped directly onto the CFD mesh at the inlet patch and is instead interpolated, there remains room for interpolation errors in the inlet conditions, which may account for some of the discrepancies that remain between the 4D-Flow MRI and CFD data.

The CFD inlet conditions used from all four spatial resolutions appear to have started to converge on a mean velocity over the patch. Over systole, the difference in mean velocity between the $4\,{\text {mm}} \times 4\,{\text {mm}} \times 4\,{\text {mm}}$ and the $3\,{\text {mm}} \times 3\,{\text {mm}} \times 3\,{\text {mm}}$ is $22.77\%$, reducing to $16.70\%$ between the $3\,{\text {mm}} \times 3\,{\text {mm}} \times 3\,{\text {mm}}$ and the $2\,{\text {mm}} \times 2\,{\text {mm}} \times 2\,{\text {mm}}$, and further reducing to $1.607\%$ between the $2\,{\text {mm}} \times 2\,{\text {mm}} \times 2\,{\text {mm}}$ and the $1.5\,{\text {mm}} \times 1.5\,{\text {mm}} \times 1.5\,{\text {mm}}$. This trend is also seen across diastole, with the differences between the resolutions being $16.17\%$, $13.92\%$, and $1.575\%$ respectively. Despite this trend, as the results have yet to fully converge on a solution, it is clear that results from the finest spatial resolution must be taken cautiously and no clinical conclusions may be made from the subsequent patient-specific CFD simulations, as it is likely they are still affected by insufficient 4D-Flow MRI resolution. It is also clear that the variability that is present within the 4D-Flow MRI data is found within the CFD simulations, suggesting that any discrepancy present in the 4D-Flow MRI data that stems from insufficient resolution will be present in any subsequent CFD results. It must be concluded therefore that agreement between 4D-Flow MRI results and CFD results does not imply accurate results.

Results from 4D-Flow MRI velocity magnitude contours indicate that a coarse spatial resolution will underestimate the velocity magnitude of the flow, whilst a finer spatial resolution will show higher a velocity magnitude in the thoracic aorta. This trend is also seen in the subsequent patient-specific CFD simulations, excluding the $4\,{\text {mm}} \times 4\,{\text {mm}} \times 4\,{\text {mm}}$ spatial resolution, which appears to erroneously predict higher velocity magnitudes alongside considerably different flow patterns. It can also be seen that CFD results appear to overestimate the velocity when compared to the 4D-Flow MRI results.

Qualitatively, the flow patterns present in the ascending aorta show better agreement at systole between the 4D-Flow MRI data and CFD results than at diastole. It is likely that this is a consequence of the inlet conditions being applied. During systole, the aortic valve opens causing a distinct and sharp flow profile, close to a parabolic profile, to be present at the inlet location. During the diastolic phase of the cardiac cycle, the aortic valve closes: there is no longer a distinct flow profile at the inlet. The fluctuations in the flow profile that are present at the inlet location during diastole are smaller in magnitude and more numerous than during systole, so are harder to capture accurately through the fit that it modelled on the 4D-Flow MRI data. This results in an inlet condition that may inaccurately model the small fluctuations during diastole, causing incorrect flow patterns to be modelled within the ascending aorta. It is also likely that insufficient velocity encoding during the 4D-Flow MRI scan is contributing to the disagreement seen at diastole.

Some of the variations that are present must be attributed to the natural variation within the patients heartbeat. Healthy cardiovascular systems are known to have complex and non-linear variability patterns that can be described by mathematical chaos, and based on the acquisition times of the 4D-Flow MRI scans, the 24 h and short-term ($\sim$ 5 min) Heart Rate Variability (HRV) of the patient must be considered^36^. The circadian rhythm, alongside core body temperature, metabolism and the sleep cycle are known to influence the 24 h variability in blood pressure and heart rate of the patient^36,37^, whilst the respiration rate is also known to influence the heart rate. Values obtained during normal breathing and paced breathing can vary significantly^38^. As the 4D-Flow MRI data is based on four separate acquisitions, it is likely not all four heartbeats will present identical haemodynamics despite all being average representations. Therefore small variations between the spatial resolutions are expected.

Although the general trend seen in the velocity results suggests that as the spatial resolution is refined, the agreement between the 4D-Flow MRI data and the CFD results improves, the $1.5\,{\text {mm}} \times 1.5\,{\text {mm}} \times 1.5\,{\text {mm}}$ spatial resolution is an exception. The differences between the 4D-Flow MRI and CFD results increase slightly from the $2\,{\text {mm}} \times 2\,{\text {mm}} \times 2\,{\text {mm}}$ spatial resolution, whilst still remaining an improvement from the $3\,{\text {mm}} \times 3\,{\text {mm}} \times 3\,{\text {mm}}$ spatial resolution. This is likely a result of the temporal resolution used for the $1.5\,{\text {mm}} \times 1.5\,{\text {mm}} \times 1.5\,{\text {mm}}$ case being coarser than that used for the remaining spatial resolutions. The impacts of the coarser temporal resolution can be seen clearly when compared the mean velocities calculated from CFD data across the axial plane in the mid-ascending aorta. There is a $6.639\%$ difference between the values reported for the $4\,{\text {mm}} \times 4\,{\text {mm}} \times 4\,{\text {mm}}$ and the $3\,{\text {mm}} \times 3\,{\text {mm}} \times 3\,{\text {mm}}$ scan, reducing to only $2.456\%$ between the $3\,{\text {mm}} \times 3\,{\text {mm}} \times 3\,{\text {mm}}$ and the $2\,{\text {mm}} \times 2\,{\text {mm}} \times 2\,{\text {mm}}$ scans. However, between the $2\,{\text {mm}} \times 2\,{\text {mm}} \times 2\,{\text {mm}}$ and the $1.5\,{\text {mm}} \times 1.5\,{\text {mm}} \times 1.5\,{\text {mm}}$, the difference rises to $17.46\%$, likely due to the increased temporal resolution.

The WSS results from CFD simulations do not follow the same trend that is present within the 4D-Flow MRI results that as the spatial resolution is increased, the WSS magnitude increases at each of the eight locations in the plane of interest. However, this behaviour in the 4D-Flow MRI data is to be expected, as it is well known that coarse spatial resolutions underestimate the WSS, as stated in multiple studies^10,11,15,16^. This suggests that the $4\,{\text {mm}} \times 4\,{\text {mm}} \times 4\,{\text {mm}}$, $3\,{\text {mm}} \times 3\,{\text {mm}} \times 3\,{\text {mm}}$, and $2\,{\text {mm}} \times 2\,{\text {mm}} \times 2\,{\text {mm}}$ may be underestimating the WSS magnitude, rather than the corresponding CFD results overestimating the WSS magnitude. The results for the 4D-Flow MRI data at both systole and diastole therefore support the conclusions drawn from these studies. This underestimation of WSS is likely causing the coarser 4D-Flow MRI spatial resolutions to not predict the region of elevated WSS in the posterior and left-posterior regions at systole that the $1.5\,{\text {mm}} \times 1.5\,{\text {mm}} \times 1.5\,{\text {mm}}$ spatial resolution and the CFD results have all indicated is present. Because it is known that WSS is systematically underestimated by 4D-Flow MRI and therefore there is no ’gold-standard’ 4D-Flow MRI results to compare the CFD data to, it is not possible to say if the larger values of WSS calculated by the patient-specific CFD simulations are erroneous, or the true values.

Despite the 4D-Flow MRI data and CFD results not following the same trend, due to the increasing magnitude of the WSS with spatial resolution, the agreement between the two methods improves as the spatial resolution increases, to the extent that the differences between the 4D-Flow MRI data and CFD results at both systole and diastole can be said to be insignificant ($p>0.05$) for the finest spatial resolution (Wilcoxon signed rank test, $\alpha =0.05$). The variability of the 4D-Flow MRI data is also much greater than that of the CFD results.

### Limitations

Although the impacts of spatial resolution have been investigated in this study, the impacts of temporal resolution have been neglected. It is likely that the temporal resolution of 4D-Flow MRI scans will also have a large influence on the data, and any subsequent CFD simulations. In order to fully assess the accuracy of 4D-Flow MRI data the temporal resolution must be investigated independently from the spatial resolution. The temporal resolution used in this study was not consistent across all four 4D-Flow MRI acquisitions. This was due to processing power limitations of the 4D-Flow MRI scanner. This will have influenced the results of the $1.5\,{\text {mm}} \times 1.5\,{\text {mm}} \times 1.5\,{\text {mm}}$ spatial resolution scan, and produced results that were less accurate than they otherwise would have been had the same temporal resolution been used. Because of this it is not possible to attribute the differences between the coarser scans and the 1.5 mm scan to the changes in spatial resolution alone.

All scans and simulations were conducted on a healthy adult patient. Because of this any recommendations as to the appropriate scan resolution needed for conducting patient-specific CFD simulations applies solely to healthy adult patients. It is not yet known how the spatial resolution will influence paediatric or neonatal simulations, or patients with existing heart conditions, where the vessel is considerably smaller or the blood flow is abnormal. It is highly likely the recommendations would differ for a paediatric or neonatal patient due to the decrease in the size of the vessel of interest.

The research was conducted on one patient only. In order to confirm the conclusions are robust, multiple patients must be investigated to ensure the trends seen are not anomalous. In the CFD simulations conducted, blood was assumed to be a Newtonian, homogeneous and incompressible fluid. In order to improve the accuracy of the simulations these assumptions should be removed. The supra-aortic vessels were neglected in this study, as in the coarse resolution scans they consisted of too few voxels to describe the flow^3^. Because of this the decision was made to remove them for all cases for consistency. Another limitation is the outlet boundary condition: a zero-pressure condition was applied to the inferior end of the descending thoracic aorta. A more physiologically accurate boundary condition, such as a three-element Windkessel model, would improve the haemodynamics within the descending aorta. As the plane of interest was more than five diameters upstream of the outlet, a zero-pressure boundary condition was considered a suitable assumption^33^. However, the authors acknowledge it to be a notable limitation to this study.

The vessel walls were assumed to be rigid. However, the thoracic aorta is known to move radially as well as vertically with every heartbeat. To account for this movement, FSI must be included in the CFD simulations. The effects of the vessel wall being impacted by the blood flow and in turn impacting the blood flow itself, the surrounding tissue and the tethering of the aorta through the intercostal, bronchial, and oesophageal arteries all play a part in the blood flow experienced through the thoracic aorta. However, a decision to neglect all FSI was made in an attempt to reduce the computational cost of the simulations.

### Future work

The temporal resolution of the 4D-Flow MRI scans was not within the scope of this investigation, and its impacts on the $1.5\,{\text {mm}} \times 1.5\,{\text {mm}} \times 1.5\,{\text {mm}}$ spatial resolution scan not investigated. Any future work into the impacts of resolution on patient-specific CFD simulations should investigate the influence temporal resolution has on subsequent CFD simulation results.

Due to the large differences in velocity and WSS present between the varying scan resolutions, the true values for the flow parameters cannot be stated as of yet. The patient must undergo further 4D-Flow MRI scans of increasingly fine spatial resolutions in order to find the point at which the results converge. This is currently limited by the processing power of the 4D-Flow MRI scanners available.

## Conclusions

It has been demonstrated that the spatial resolution the 4D-Flow MRI scan is acquired at has major consequences on subsequent patient-specific CFD simulations that are undertaken in terms of the volumetric flow rate, the vessel diameter, the velocity, and the WSS. The differences that arise between CFD simulations based on various resolutions have been established; refining the spatial resolution of a 4D-Flow MRI scan from $4\,{\text {mm}} \times 4\,{\text {mm}} \times 4\,{\text {mm}}$ to $1.5\,{\text {mm}} \times 1.5\,{\text {mm}} \times 1.5\,{\text {mm}}$ produces a difference in the mean velocity magnitude experienced in patient-specific CFD simulations of $23.23\%$ and $21.42\%$ at systole and diastole respectively. It has been shown that there is a considerable lack of consistency during the systolic and diastolic phases of the cardiac cycle in the results when comparing the various spatial resolutions, in both 4D-Flow MRI data and the CFD data.

The results presented within this study show that 4D-Flow MRI spatial resolutions of $4\,{\text {mm}} \times 4\,{\text {mm}} \times 4\,{\text {mm}}$, $3\,{\text {mm}} \times 3\,{\text {mm}} \times 3\,{\text {mm}}$, and $2\,{\text {mm}} \times 2\,{\text {mm}} \times 2\,{\text {mm}}$ are wholly unsuitable for use in patient-specific CFD simulations. It has been shown that an insufficient resolution produces poor agreement between 4D-Flow MRI data and CFD results, in addition to poor agreement with results from an increased spatial resolution. However, a $1.5\,{\text {mm}} \times 1.5\,{\text {mm}} \times 1.5\,{\text {mm}}$ spatial resolution cannot be recommended for use without caution. As results have not yet converged on a solution, it is not known the degree of error that is present in a patient-specific CFD simulation that is bas.ed on 4D-Flow MRI data acquired with a spatial resolution of $1.5\,{\text {mm}} \times 1.5\,{\text {mm}} \times 1.5\,{\text {mm}}$Caution must also be advised with the results from the $1.5\,{\text {mm}} \times 1.5\,{\text {mm}} \times 1.5\,{\text {mm}}$spatial resolution due to temporal resolution that was used to acquire the 4D-Flow MRI data.

Based on the results presented in this study, the authors recommend that when 4D-Flow MRI scan data is used to construct and run patient-specific CFD simulations on healthy adult patients, a minimum spatial resolution of $1.5\,{\text {mm}} \times 1.5\,{\text {mm}} \times 1.5\,{\text {mm}}$ should be used to avoid inaccurate data being used. A spatial resolution coarser than $1.5\,{\text {mm}} \times 1.5\,{\text {mm}} \times 1.5\,{\text {mm}}$will generate results with substantial distortions that greatly underestimate the magnitude of the velocity within the thoracic aorta, as well as produce differences in terms of the geometry, the volumetric flow rate, and the WSS. As this study does not investigate the impacts of 4D-Flow MRI resolution on patient-specific CFD studies of diseased aortas, recommendations cannot be made explicitly regarding a spatial resolution that would be appropriate. However, as the presence of disease or heart defects results in more complex flow features, it is likely that a spatial resolution of $1.5\,{\text {mm}} \times 1.5\,{\text {mm}} \times 1.5\,{\text {mm}}$ must be the minimum used, and if possible a finer resolution be utilised to avoid any complex flow features being neglected.

As CFD is quickly becoming an invaluable tool in the medical field, there is a drive towards using it to aid in treatment planning, diagnostics, monitoring disease progression and risk stratification. When this is the case, the authors of this study advise great caution if using inappropriate spatial resolutions as any miscalculation as a result of resolution may lead to misleading or inaccurate results being passed onto clinicians, which may have serious consequences for the patient in question.

## Data Availability

The data-sets generated and analysed in this study are not publicly available as this may compromise the individuals privacy. Data may be available from the corresponding author upon reasonable request.
